# Temperature effects on sinking velocity of different *Emiliania huxleyi* strains

**DOI:** 10.1371/journal.pone.0194386

**Published:** 2018-03-20

**Authors:** Anaid Rosas-Navarro, Gerald Langer, Patrizia Ziveri

**Affiliations:** 1 Institute of Environmental Science and Technology (ICTA), Autonomous University of Barcelona (UAB), Bellaterra, Barcelona, Spain; 2 The Marine Biological Association of the United Kingdom, The Laboratory, Citadel Hill, Plymouth, Devon, PL1 2PB, United Kingdom; 3 Catalan Institution for Research and Advanced Studies (ICREA), Barcelona, Spain; Oregon State University, UNITED STATES

## Abstract

The sinking properties of three strains of *Emiliania huxleyi* in response to temperature changes were examined. We used a recently proposed approach to calculate sinking velocities from coccosphere architecture, which has the advantage to be applicable not only to culture samples, but also to field samples including fossil material. Our data show that temperature in the sub-optimal range impacts sinking velocity of *E*. *huxleyi*. This response is widespread among strains isolated in different locations and moreover comparatively predictable, as indicated by the similar slopes of the linear regressions. Sinking velocity was positively correlated to temperature as well as individual cell PIC/POC over the sub-optimum to optimum temperature range in all strains. In the context of climate change our data point to an important influence of global warming on sinking velocities. It has recently been shown that seawater acidification has no effect on sinking velocity of a Mediterranean *E*. *huxleyi* strain, while nutrient limitation seems to have a small negative effect on sinking velocity. Given that warming, acidification, and lowered nutrient availability will occur simultaneously under climate change scenarios, the question is what the net effect of different influential factors will be. For example, will the effects of warming and nutrient limitation cancel? This question cannot be answered conclusively but analyses of field samples in addition to laboratory culture studies will improve predictions because in field samples multi-factor influences and even evolutionary changes are not excluded. As mentioned above, the approach of determining sinking rate followed here is applicable to field samples. Future studies could use it to analyse not only seasonal and geographic patterns but also changes in sinking velocity over geological time scales.

## Introduction

It is generally acknowledged that global climate change will impact marine phytoplankton in terms of physiology. These physiological changes are usually considered in isolation. However, physiological changes might also have secondary effects, which can themselves feedback on physiology. An example of such a secondary effect is sinking velocity, which influences the position in the water column in which the cells reside. Sinking of the biogeochemically important coccolithophores is strongly influenced by their calcareous shell, the coccosphere. The comparatively high density of calcite makes the coccosphere act as ballast stones [1–3]. Although surface ocean mixing rates are high compared to coccolithophore sinking rates, some of the bigger species such as *Coccolithus pelagicus* are exported as individual cells [4–7]. This is counter intuitive because the sinking velocity of e.g. *C*. *pelagicus* is ca. 6 m/d [8] and slow compared to surface ocean mixing rates of ca. 100 m/d [9]. Calculations combining *E*. *huxleyi* growth rates, sinking rates, and surface ocean mixing rates suggest that individual cell sinking in this species is relatively unimportant whereas it might be relevant in heavier species such as *C*. *pelagicus* and *Calcidiscus leptoporus* [7]. However, our choice of *E*. *huxleyi* was not primarily motivated by the ecological relevance of its sinking. We chose *E*. *huxleyi* to make this study maximally comparable to previous studies using the same equations to calculate individual cell density and sinking rate (see below).

Moreover, climate change will result in a more pronounced stratification of the water column, thereby increasing the significance of sinking. Stratification will also lead to nutrient depletion in the photic zone where coccolithophores thrive. It has traditionally been assumed that nutrient limitation enhances calcification, and increases sinking velocities [9–12]. This mechanism was regarded as a means to reach deeper, nutrient rich water layers. The slim, currently available, evidence however, does not support this view [3, 8, 13].

Climate change induced nutrient depletion will be accompanied by seawater acidification and a rise in temperature [14–18]. Hence, in order to assess future sinking behaviour of coccolithophores, these factors also have to be considered. A recent study analysed sinking velocities of *E*. *huxleyi* in response to acidification and temperature increase [19]. While acidification had no effect on sinking velocity, a temperature increase had a profound effect, increasing sinking velocity. This suggests that in the context of climate change temperature might be an important factor influencing sinking velocity. However, the study by Milner et al. [19] is of somewhat limited evidential strength, because only one strain of *E*. *huxleyi* was analysed at two different temperatures. Moreover, the temperature optimum for this strain is unknown, although it is fair to assume that the sub-optimal temperature range was studied [19]. In this study we analyse the sinking velocity response to temperature of three further *E*. *huxleyi* strains in the sub-optimal temperature range. In the context of global climate change, the sub-optimal temperature range is of particular interest because global warming over the next century will result in surface ocean temperatures which are still sub-optimal for most coccolithophore clones [20–23]. In terms of cell physiology the distinction between sub-optimal and supra-optimal temperatures is important because the latter usually have dramatic effects which can be attributed to heat-damage of e.g. proteins [24, 25]. Sub-optimal temperatures, by contrast, generally produce less obvious effects and it is by no means straightforward to predict whether an effect will be observable or not [23]. That is why we analysed sinking velocities in response to sub-optimum to optimum temperatures here. Our analyses are not based on direct measurements of sinking rate. Instead, we employ a recently introduced method of calculating sinking rates from coccosphere architecture [26]. This approach yields *E*. *huxleyi* sinking rates which tally well with directly measured rates, and has the advantage of being applicable to culture samples and field samples including fossil material. Hence there is the potential for comparative culture-field studies. The first calculations by Hoffmann et al. [26] were based on FIB-SEM data, the production of which is technically difficult and time consuming. Subsequent studies, however, used data based on conventional SEM, light microscopy, and cell counters, which are readily available in the framework of a culture experiment [8, 19]. We followed this latter approach to make our data and calculations comparable to the ones by Milner et al. [19], because it was the aim of this study to build upon their initial sinking rate-temperature data. We used three different *E*. *huxleyi* strains to test for strain specificity, and four different temperatures, the highest of which representing the optimum temperature of these strains.

## Materials and methods

With the main objective of calculating the sinking velocity of individual cells of three strains of *Emiliania huxleyi* grown at different temperatures, under non-limiting conditions of light and nutrients, we used the Stokes’ law [26]. To use the Stokes’ law formula the following four measurements were needed: number of observed attached coccoliths, coccosphere diameter, protoplast diameter, and coccolith calcite mass. The first two were measured from scanning electronic microscopy (SEM) images and the protoplast diameter with a Coulter Counter. The coccolith calcite mass variable results of the experiment are already analysed by Rosas-Navarro et al. [23]. Temperature and salinity were necessary to calculate the water density and the dynamic viscosity of water, both required variables to calculate the sinking velocity. The experiment was performed by triplicate. In the following subsections we provide a full description of the whole experiment and calculations.

Another objective of the present study was to compare the particulate inorganic carbon (PIC) in individual cells (that is the PIC in the attached coccoliths) with the bulk PIC (that is the PIC in both attached and detached coccoliths). The bulk PIC was chemically derived and was analysed by Rosas-Navarro et al. [23].

We also calculated a geometrically derived particulate organic carbon (POC) and compared it with the chemically derived POC; this last one was analysed by Rosas-Navarro et al. [23]. Similarly, we calculated and compared individual PIC: POC ratios using the individual PIC and the geometrically and chemically derived results for POC. We compared the individual PIC: POC with the bulk PIC: POC. Throughout the text we use the word "individual" to make clear that the PIC or calcite of the attached coccoliths was used, as opposed to bulk PIC including loose coccoliths.

### Pre-culture and batch culture experiments

Clonal cultures of *Emiliania huxleyi* were obtained from the Roscoff Culture Collection. We selected three strains of *E*. *huxleyi*, two from the Japanese coast in the North Pacific Ocean (RCC1710 –synonym of NG1 and RCC1252 –synonym of AC678 and MT0610E) and a third strain from the same region but of unknown exact origin and strain name, named here IAN01. Strain RCC1710 was collected off Nagasaki at Tsushima Strait (Japan) and RCC1252 at Tsugaru Strait (Japan); both places are strongly influenced by the Tsushima warm current. Additional information about the strain RCC1252 can be found at http://roscoff-culture-collection.org/.

The culture media was sterile-filtered North Sea water (filtered through 0.2 μm pore size sterile Sartobran 300 filter cartridges, Sartorius, Germany) supplemented with nutrients (nitrate -882.5 Μm- and phosphate -36.25 μM), metals and vitamins according to Guillard and Ryther [27]. Cell densities, called here cell concentration to prevent confusion with the individual density, and cell diameter were determined using a Multisizer 3 Coulter Counter (Beckman Coulter for particle characterization). To prevent significant changes in seawater carbonate chemistry, maximum cell concentrations were limited to ≈ 1×10^5^ cells ml^-1^ (e.g. [28]). We used a 16/8 light/dark cycle, and an irradiance of ≈ 300 μmol photons s^-1^ m^-2^ in an incubator, where transparent culture flasks during the acclimation, and latter the transparent glass bottles during the experiment, were located in the incubator so that they would not block the path of light. For acclimation to the different temperatures of the experiment before harvesting, the three strains were grown at the different temperatures in 200 ml culture media solution in polycarbonate culture flasks of 250 ml volume, at initial densities of 4000 cells per millilitre, with daily observation and quantification for at least 20 generations. The frequency of inoculation in new flasks varied depending on the cell concentration or the amount of water remaining (since it was subtracted for the measurements) so therefore on each growth rate.

The dilute batch culture experiments were conducted in triplicate, i.e. in three different incubation bottles, for the strains RCC1710 and RCC1252 at 10, 15, 20 and 25°C of temperature, and for IAN01 at 15, 20 and 25°C. The strains were grown in 2 L of sea water within transparent sterilized 2.3 L glass bottles. Cell concentration at inoculation was 500 to 1000 cells ml^-1^ and at harvest it was a maximum of ≈ 1×10^5^. Harvesting was done 9 h after the onset of the light period, lasting between 1 and two hours.

### Coccosphere’s number of attached coccoliths and diameter

Thirty millilitres of culture was filtered onto polycarbonate filters (0.8 μm pore size) and dried at 60°C for 24 h. A small portion (~ 0.7 cm^2^) of each filter was mounted on an aluminium stub and coated with gold (EMITECH K550X sputter coater). Images were captured along random transects using a ZEISS-EVO MA10 SEM.

The SEM images were used to analyse ~ 50 complete coccospheres per sample by counting the number of attached coccoliths observed in each coccosphere (including those observed below other coccoliths in multi-layered coccospheres). The number of attached coccoliths per coccosphere was estimated dividing the number of visible attached coccoliths per coccosphere by 0.75 [26].

As coccospheres were mostly oval and in some cases irregular, their diameter was calculated using the surface area of the coccosphere in the formula for area of a circle. The surface area of the coccospheres was manually measured on the SEM images using the program ImageJ. Measurements in pixels were transformed to micrometres according to the corresponding scale of the SEM images.

### Individual density and sinking velocity

Individual density was estimated dividing the total individual mass by the total individual volume [26]. Cell (protoplast) diameter was recorded from the Multisizer 3 Coulter Counter data and was used to calculate the cell volume and to estimate the cell mass assuming a density of 1.05 g cm^3^ for the organic cell matter [29]. Coccosphere calcite mass was calculated as the product of coccolith mass (results from [23]) by the number of attached coccoliths per cell. Coccosphere calcite volume was estimated assuming a density for calcite of 2.7 g cm^3^ [29]. Total individual volume was calculated from the measured coccosphere diameter. For the total individual mass were considered the spaces between coccoliths presumably filled with seawater. Hence, volume of seawater was calculated from the difference of the total individual volume minus the sum of the volumes of the organic (protoplast) plus the inorganic (calcite) cellular components. Seawater mass was estimated from a calculated seawater density for each temperature (Table 1). Seawater density was calculated for each temperature treatment, for a measured salinity of 32 ‰ and at atmospheric pressure, according to Millero et al. [30]. Therefore, individual density was estimated dividing the sum of the three masses (protoplast, calcite, and seawater) by the total volume estimated from the measured coccosphere diameter.

**Table 1:** Seawater density and seawater dynamic viscosity.

*T*	Water density	Dynamic viscosity
[°C]	[g cm^-3^]	[g cm^-1^ s^-1^]
10	1.025	0.014
15	1.024	0.012
20	1.022	0.011
25	1.021	0.010

Values in Table 1 were used for the estimation of the individual sinking velocity at the different temperature (*T*) treatments.

According to Stokes’ law, individual sinking velocity was calculated according to:

$$v_{s}=\frac{2(\rho_{i}-\rho_{sw})∙g∙R^{2}}{9∙v_{sw}},$$ (1)

where *v*_s_ is the individual sinking velocity (in m d^-1^) (vertically downwards if ρ_i_ > ρ_sw_), *g* is the gravitational acceleration (in m d^-2^), *R* is the radius of the coccosphere (in m), ρ_i_ is the individual density (in g m^-3^), ρ_sw_ is seawater’s density (in g m^-3^) and *v*_sw_ is the dynamic viscosity of seawater (in g m^-1^ d^-1^). Dynamic viscosity was calculated for each temperature (Table 1), for a salinity of 32‰ and at atmospheric pressure, according to Sharqawy et al. [31].

### Geometrically derived PIC in individual cells

Particulate inorganic carbon in individual cells, i.e. PIC in the attached coccoliths per coccosphere, was calculated using SEM and light microscopy (LM) results. It was calculated using the measured PIC per coccolith derived from LM coccolith mass measurements [23], and the number of attached coccoliths per coccosphere obtained from SEM counts.

Geometrically derived PIC in individual cells was then calculated as follows:

$${PIC}_{{cell}^{-1}}=\frac{M_{c}∙12.0107∙C_{{sph}^{-1}}}{100.0869},$$ (2)

where ${PIC}_{{cell}^{-1}}$ = cellular PIC (in pg), *M*_c_ = coccolith calcite mass (in pg), 12.0107 corresponds to the relative atomic mass of carbon, 100.0869 corresponds to the relative molecular mass of calcite, $C_{{sph}^{-1}}$ = attached coccoliths per coccosphere. Calculations were made for each replicate.

### Theoretical number of detached coccoliths

The theoretical number of detached coccoliths per cell and the loose PIC per cell were calculated using the chemically derived bulk PIC per cell, the PIC per coccolith measured through microscopy techniques (both variables from the results published by Rosas-Navarro et al. [23]), and the counted number of attached coccoliths per coccosphere. The calculation for the theoretical number of detached coccoliths per cell was: total coccoliths per cell (from chemically measured cellular PIC divided by the LM derived PIC per coccolith) minus attached coccoliths per coccosphere (from SEM counts). We got the loose PIC per cell multiplying the number of detached coccoliths per cell by the measured PIC per coccolith. The calculations were done for each replicate.

### Geometrically derived cellular POC

Geometrically derived cellular POC quota was calculated following Menden-Deuer and Lessard [32] as in Hoffmann et al. [26], according to the following equation:

$${POC}_{{cell}^{-1}}=0.216∙{V_{cell}}^{0.939},$$ (3)

where POC_cell_^-1^ = cellular POC (in pg), *V*_cell_ = cell (protoplast) volume (in μm^3^) calculated from the cell diameter measured with the Coulter Counter, 0.216 and 0.939 correspond to constants for plankton [26, 32]. Calculations were made for each replicate.

The PIC: POC ratio for individual cells, i.e. only considering the attached coccoliths and not the detached coccoliths, was calculated using the geometrically derived cellular PIC quota in the ratio instead of the chemically derived PIC quota. A purely geometrically derived PIC: POC ratio was also calculated using the geometrically derived cellular PIC and POC quotas.

### Statistics

For the three *E*. *huxleyi* strains together, ANOVA (two-factor with replication) was used to test whether a response variable (e.g. individual sinking velocity) presented significant (*p* < 0.05) differences between the temperature treatments, to test whether the effect was strain-independent or strain-specific (*p* < 0.05), and to test whether there were significant differences in the interaction between treatment and strain (*p* < 0.05) and therefore whether the different strains respond similarly or not regardless of whether they were presenting differences between them. Degrees of freedom are given as subscripts of *F*. For the different methods used for calculating the cellular POC and the individual cell PIC: POC ratio, a t-Test (two-tail) was used to test the null hypothesis that the means of the two methods are equal (*alpha* = 0.05).

Each measured variable, for each of the tripled bottles, came from a statistically big number of samples (of ~ 50 for the case of SEM samples, of minimum 300 for the LM samples, and of ~ 50 000 for the Coulter Counter samples), reducing the standard errors and therefore strengthening all the statistical analysis done. Except for the regressions reported in the figures which used the mean values of the triplicates, the rest of statistical analysis used the values of each replicate. In the results we report the means and the standard deviations of the triplicates. For the case of the calculated individual sinking velocity, to strengthen the results due to the involvement of several variables each one with its own standard error, we calculated the error propagation of the individual sinking velocity calculation for each replicate (three replicates per temperature treatment and strain). The errors used for the error propagation calculation were the standard errors of the four measured variables (number of observed attached coccoliths, protoplast diameter, coccolith calcite mass, and coccosphere diameter), the errors of the variables water density and dynamic viscosity which were calculated using the errors derived from the maximum variation found for temperature and salinity during the experiment (0.5°C and 0.5‰ respectively), and the error for the gravitational acceleration based on the maximum and minimum values that can be found on Earth. These results are more detailed in the supporting information.

## Results

### Coccosphere morphology and mass, and cell diameter

**Fig 1:**
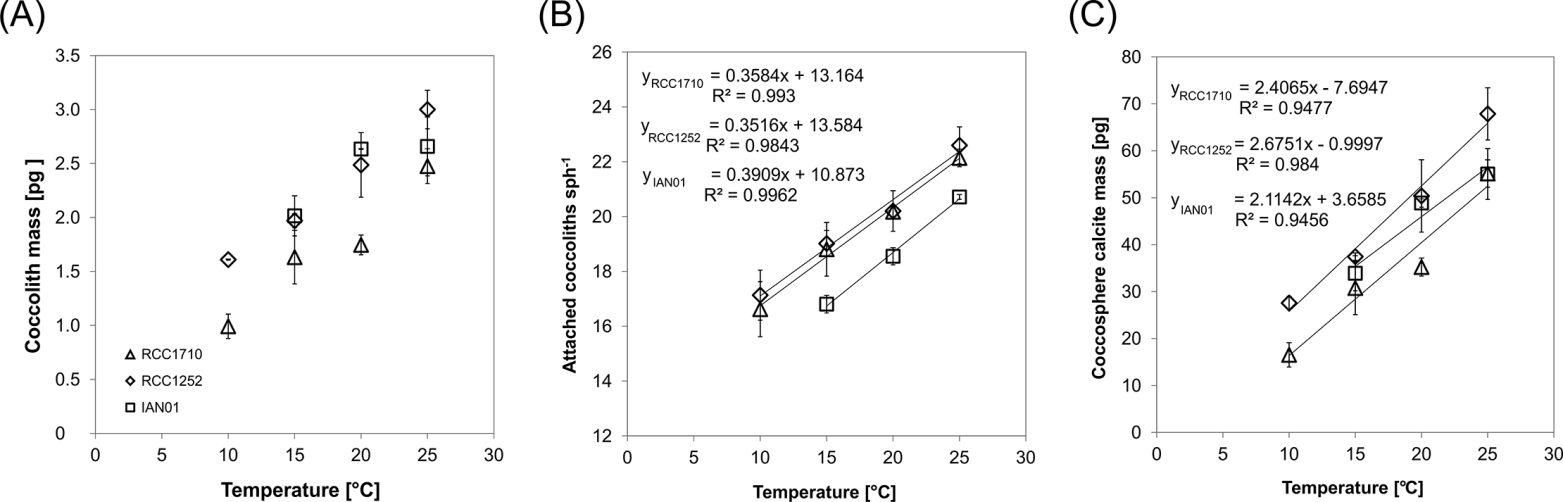
Temperature effect in the coccosphere calcite mass. The coccosphere calcite mass (C) was calculated from the coccolith mass (A) and the number of attached coccoliths per coccosphere (B). Standard deviations of the triplicate experiment results are shown. Linear trend lines, corresponding equations, and r-squared values are shown in Figs B and C. The following three different strains of *E*. *huxleyi* were used: RCC1710 (triangle symbols), RCC1252 (diamond symbols), and IAN01 (square symbols). Fig A is from Rosas-Navarro et al. [23].

Independently of the strain, the number of attached coccoliths per coccosphere increased linearly with temperature (Fig 1B and Table 2). There were no significant differences in the interaction between treatment and strain of the three strains (*F*_4_ = 0.37, *p* = 0.824), so there were no significant differences between their slopes, even though the number of attached coccoliths of the strain IAN01 was lower than that of the other two strains. There were no significant differences between strains RCC1710 and RCC1252 (*F*_1_ = 1.05, *p* = 0.321). On average, the number of attached coccoliths increased with temperature ~ 5.5 coccoliths from 10 to 25°C, ~ 3.6 coccoliths from 15 to 25°C, and ~ 2.2 coccoliths from 20 to 25°C; on average, coccospheres had 1.9 coccoliths more (or 1.1 times more) each 5°C.

**Table 2:** Protoplast and coccosphere diameter, number of attached and detached coccoliths per cell, particulate inorganic carbon (PIC) per coccolith, and loose PIC per cell.

Strain	*T*	Prot diam	Sph diam	Att liths	Det liths	PIC/lith	Loose PIC/cell
	[°C]	[μm]	[μm]	sph^-1^	cell^-1^	[pg]	[pg]
RCC1710	10	4.80 (0.01)	6.44 (0.16)	16.62 (1.00)	103.87 (2.84)	0.12 (0.01)	13.16 (0.06)
RCC1710	15	5.24 (0.17)	6.91 (0.22)	18.81 (0.98)	54.35 (14.48)	0.20 (0.03)	10.29 (0.54)
RCC1710	20	4.99 (0.01)	6.98 (0.26)	20.17 (0.23)	32.99 (0.57)	0.21 (0.01)	7.12 (0.03)
RCC1710	25	4.78 (0.06)	6.95 (0.07)	22.14 (0.32)	13.79 (1.26)	0.30 (0.02)	4.09 (0.11)
RCC1252	10	4.60 (0.05)	6.27 (0.39)	17.13 (0.91)	25.79 (2.64)	0.19 (0.00)	4.98 (0.52)
RCC1252	15	4.86 (0.03)	6.75 (0.14)	19.02 (0.48)	22.97 (0.70)	0.24 (0.01)	5.43 (0.23)
RCC1252	20	4.47 (0.02)	6.82 (0.19)	20.20 (0.74)	14.63 (7.36)	0.30 (0.04)	4.06 (1.47)
RCC1252	25	4.89 (0.00)	7.51 (0.07)	22.60 (0.67)	11.38 (3.00)	0.36 (0.02)	4.06 (0.87)
IAN01	15	4.53 (0.05)	6.07 (0.19)	16.81 (0.32)	25.44 (3.10)	0.24 (0.02)	6.11 (0.17)
IAN01	20	4.51 (0.01)	5.92 (0.11)	18.55 (0.31)	7.13 (0.63)	0.32 (0.00)	2.26 (0.20)
IAN01	25	4.58 (0.05)	6.66 (0.38)	20.71 (0.09)	14.74 (4.70)	0.32 (0.03)	4.60 (1.00)

Standard deviation of the triplicates is shown in parentheses. Temperature (*T*); protoplast (prot); coccosphere (sph); diameter (diam); attached (att); detached (det); coccolith (lith).

Coccosphere calcite mass (Fig 1C and Table 3), calculated from coccolith mass and number of attached coccoliths per coccosphere, increased linearly from 10 to 25°C in the three strains. It presented significant differences between the treatments (*F*_2_ = 64.34, *p* = 0.000), also between the strains (*F*_2_ = 12.66, *p* = 0.000), but there were no significant differences in the interaction between treatment and strain (*F*_4_ = 2.29, *p* = 0.099). Coccosphere calcite mass increased on average 41% or 12.75 pg each 5°C.

**Table 3:** *Emiliania huxleyi* individual density and individual sinking velocity estimations and values of the variables involved.

Strain	*T*	Prot mass	Prot vol	Sph calc mass	Sph calc vol	SW mass	SW vol	Ind mass	Ind vol	Ind density	Ind sink velocity
	[°C]	[pg]	[μm^3^]	[pg]	[μm3]	[pg]	[μm3]	[pg]	[μm3]	[g cm^-3^]	[m d^-1^]
RCC1710	10	60.75 (0.29)	57.86 (0.28)	16.53 (2.59)	6.12 (0.96)	77.64 (10.15)	75.78 (9.91)	154.93 (10.66)	139.76 (10.04)	1.11 (0.01)	0.12 (0.02)
RCC1710	15	79.48 (7.70)	75.69 (7.34)	30.77 (5.68)	11.39 (2.10)	87.86 (24.25)	85.83 (23.69)	198.10 (16.60)	172.91 (16.16)	1.15 (0.02)	0.23 (0.04)
RCC1710	20	68.38 (0.49)	65.12 (0.47)	35.23 (1.91)	13.05 (0.71)	102.37 (19.30)	100.12 (18.88)	205.97 (21.70)	178.29 (20.05)	1.16 (0.01)	0.29 (0.00)
RCC1710	25	59.89 (2.43)	57.04 (2.32)	55.15 (2.91)	20.43 (1.08)	99.13 (6.89)	97.08 (6.75)	215.21 (6.29)	175.76 (5.58)	1.23 (0.02)	0.49 (0.03)
RCC1252	10	53.65 (1.57)	51.10 (1.50)	27.64 (1.47)	10.24 (0.55)	70.49 (23.85)	68.80 (23.28)	151.78 (25.94)	130.13 (24.44)	1.17 (0.02)	0.19 (0.00)
RCC1252	15	62.99 (1.20)	59.99 (1.14)	37.45 (0.79)	13.87 (0.29)	89.07 (10.29)	87.01 (10.05)	189.51 (9.99)	160.87 (10.04)	1.18 (0.01)	0.27 (0.01)
RCC1252	20	49.03 (0.67)	46.69 (0.64)	50.38 (7.71)	18.66 (2.85)	103.43 (13.08)	101.16 (12.79)	202.84 (18.16)	166.51 (14.08)	1.22 (0.02)	0.40 (0.05)
RCC1252	25	64.10 (0.14)	61.05 (0.13)	67.85 (5.56)	25.13 (2.06)	138.89 (8.10)	136.02 (7.93)	270.84 (5.14)	222.20 (6.55)	1.22 (0.02)	0.55 (0.05)
IAN01	15	51.19 (1.55)	48.75 (1.47)	33.91 (3.72)	12.56 (1.38)	57.10 (11.05)	55.78 (10.79)	142.19 (13.18)	117.09 (11.34)	1.21 (0.02)	0.27 (0.02)
IAN01	20	50.30 (0.29)	47.90 (0.28)	48.80 (0.82)	18.07 (0.30)	43.96 (6.73)	43.00 (6.58)	143.06 (5.97)	108.97 (6.33)	1.31 (0.02)	0.45 (0.02)
IAN01	25	52.91 (1.89)	50.39 (1.80)	55.05 (5.42)	20.39 (2.01)	86.71 (28.07)	84.92 (27.49)	194.67 (31.34)	155.70 (27.61)	1.25 (0.02)	0.51 (0.02)

Calculated masses and volumes involved in the estimations are shown. Standard deviation of the triplicates is shown in parentheses. Temperature (*T*); protoplast (prot); volume (vol); coccosphere (sph); calcite (calc); extracellular matrix seawater (SW); individual (ind); sinking (sink).

Coccosphere diameter results (Table 2) showed statistically significant differences between the three strains (*F*_2_ = 42.01, *p* = 0.000) but were found significant differences between the treatments in which the three strains presented smaller coccospheres at lower experimental temperatures and larger coccospheres at higher experimental temperatures.

Cell diameter (Table 2) did not show any strain-independent or strain-specific trend related with temperature or any other variable.

### Individual density and sinking velocity

**Fig 2:**
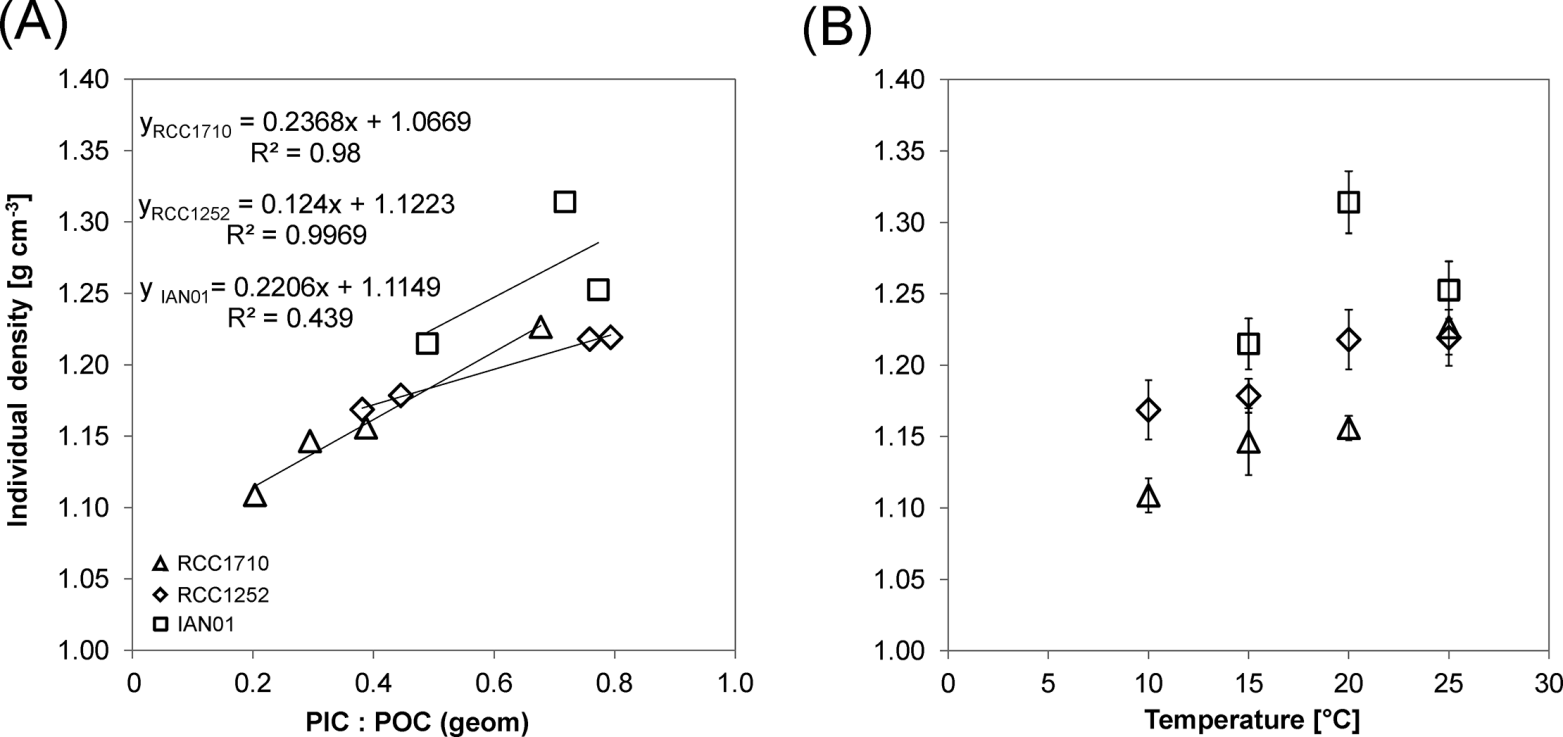
**Individual cell density versus the geometrically derived ratio between the particulate inorganic carbon and the particulate organic carbon (A) and versus temperature (B).** RCC1710 (triangle symbols), RCC1252 (diamond symbols), and IAN01 (square symbols). Linear trend lines, corresponding equations, and r-squared values are shown in Fig A.

Individual density was calculated considering the organic and inorganic components of the cell and the spaces between the coccoliths presumably filled with seawater (Table 3); total mass was divided by the individual volume which was calculated from the coccosphere diameter (Table 2). Individual density was lowest at the lowest temperature treatment independently of the strain (Fig 2B). The treatments presented significant differences (*F*_2_ = 21.71, *p* = 0.000), but there were also significant differences between the three strains and in the interaction between treatment and strain. Highest differences between intervals of 5°C, were found between 15 and 20°C in strains RCC1252 and IAN01, and between 20 and 25°C in strain RCC1710. Individual density changes were on average 2% higher each 5°C.

**Fig 3:**
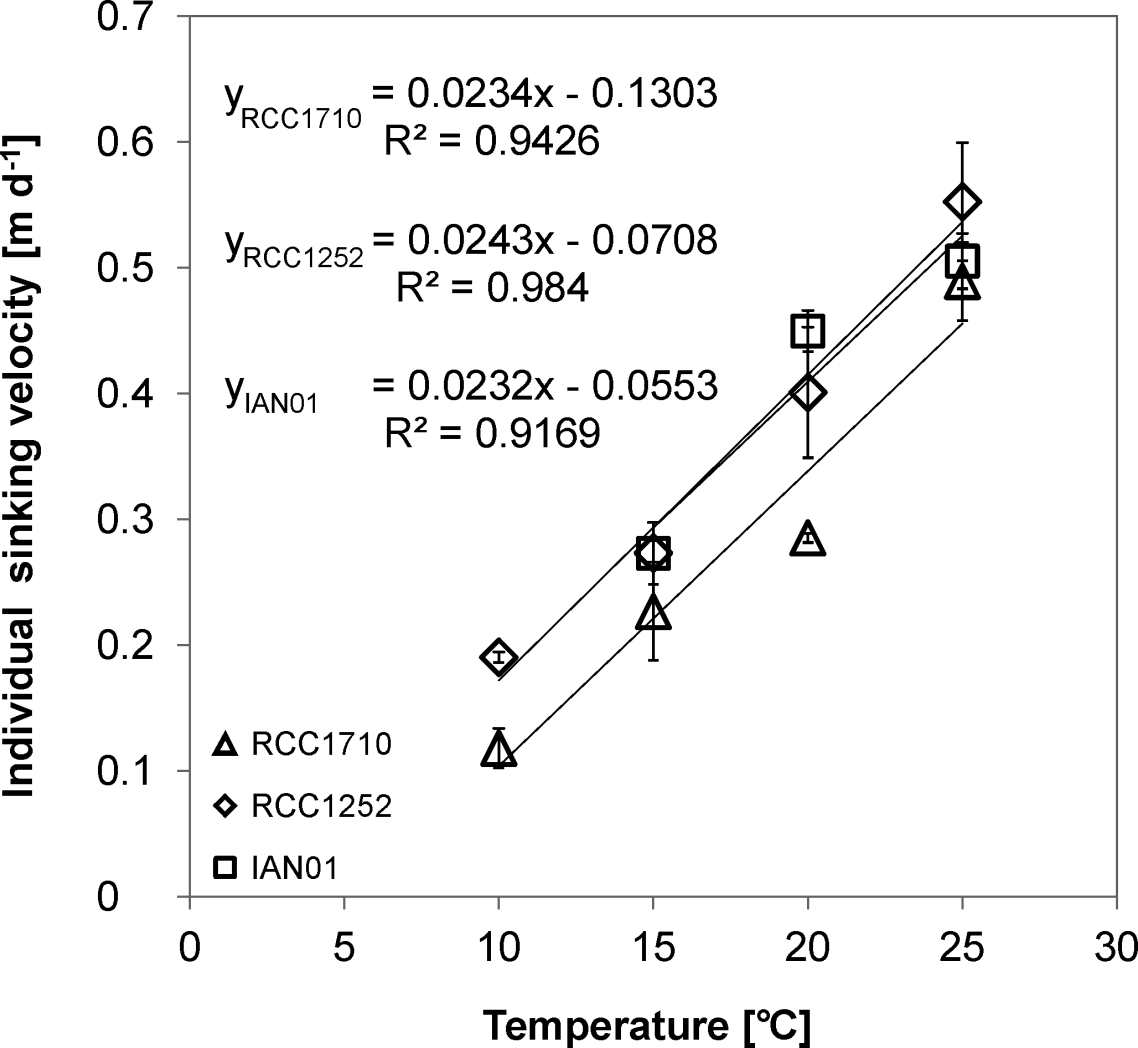
Changes in the individual sinking velocity calculated for three different strains of *E*. *huxleyi* grown at different temperatures. Strain RCC1710 (triangle symbols), strain RCC1252 (diamond symbols), and strain IAN01 (square symbols). Standard deviations of the triplicate experiment results, linear trend lines, corresponding equations, and r-squared values are shown.

Individual sinking velocity (Fig 3 and Table 3) presented significant differences between treatments (*F*_2_ = 133.45, *p* = 0.000), it was significantly positively correlated with temperature, independently of the strain, and the carbonate system variations did not improve the correlation significantly. The calculated velocities with their error propagation showed a positive trend with temperature in the three strains (see supporting information). No significant differences were found between strains RCC1252 and IAN01, neither in their interaction between treatment and strain. Strain RCC1710 presented significant differences with the other two strains but no significant difference with the strain RCC1252 in the interaction between treatment and strain (*F*_3_ = 1.43, *p* = 0.271). The increase in velocity each 5°C was of ~ 50%. Maximum individual sinking velocity in the three strains was found at 25°C and was of ~ 0.5 m d^-1^.

### Detached coccoliths and PIC

The theoretical numbers of detached coccoliths per cell (Table 2) were significantly different between the temperature treatments, between the strains and in the interaction between treatment and strain. Part of the variation could be due to detaching because of the sampling procedure, to minimize it, careful management of the samples was always taken, for example the mixing of the cultures to avoid sedimentation was always performed with a very gentle rotation and the pressure of the pump during filtration limited to 200 mbar. Maximum numbers of detached coccoliths were found at 10°C in strain RCC1710 with ~ 100 detached coccoliths per cell and minimum numbers in all strains were on average ~ 11 detached coccoliths per cell. Maximum values of loose PIC per cell were found at 10°C in strain RCC1710, they were of 13 pg per cell, and minimum values were on average of 3.5 pg per cell. The linear correlation between the loose PIC per cell (Table 2) and the number of detached coccoliths per cell had an *R* of 0.97.

### Chemically and geometrically derived PIC and POC

The geometrically derived PIC quota for individuals cells is proportional to the coccosphere calcite mass (Fig 1C) (the individual PIC gives the carbon (C) of the calcite (CaCO_3_) in the coccosphere), they increased linearly from 10 to 25°C independently of the strain.

The results obtained from the different methods used for calculating the cellular POC (geometrical or chemical, Table 4 and S2 Fig) and the individual cell PIC: POC ratio (geom:geom or geom:chem in Table 4) do not present significant differences (*t*_two-tail_ = 1.99, *df* = 61; *t*_two-tail_ = 2.00, *df* = 59, respectively). The POC ratio data (ratio calculated from the chemically derived POC divided by the geometrically derived POC) fits a normal distribution (S2 Fig) and has a mean value of 0.99 with a standard deviation of 0.05.

**Table 4:** Chemically (chem) and geometrically (geom) derived particulate inorganic carbon (PIC) and particulate organic carbon (POC).

Strain	*T* [°C]	PIC (chem)[pg cell^-1^]	PIC (geom)[pg sph^-1^]	POC (chem) [pg cell^-1^]	POC (geom) [pg cell^-1^]	PIC: POC (chem: chem)	PIC: POC (geom: geom)	PIC: POC (geom: chem)	POC ratio (chem: geom)
RCC1710	10	15.31 (0.15)	1.98 (0.31)	8.91 (0.29)	9.76 (0.04)	1.72 (0.07)	0.2 (0.03)	0.24 (0.03)	0.92 (0.03)
RCC1710	15	14.07 (0.4)	3.69 (0.68)	9.9 (0.11)	12.55 (1.14)	1.42 (0.02)	0.29 (0.05)	0.38 (0.09)	0.83 (0.04)
RCC1710	20	11.47 (0.09)	4.23 (0.23)	12.05 (0.79)	10.9 (0.07)	0.95 (0.05)	0.39 (0.02)	0.36 (0.01)	1.1 (0.07)
RCC1710	25	10.8 (0.24)	6.62 (0.35)	9.3 (0.8)	9.63 (0.37)	1.17 (0.13)	0.68 (0.06)	0.68 (0.07)	0.96 (0.05)
RCC1252	10	8.29 (0.49)	3.31 (0.17)	6.35 (0.11)	8.68 (0.24)	1.31 (0.06)	0.38 (0.02)	0.52 (0.03)	0.73 (0.02)
RCC1252	15	9.92 (0.32)	4.49 (0.1)	8.64 (0.29)	10.09 (0.18)	1.15 (0.07)	0.45 (0.01)	0.52 (0.03)	0.86 (0.04)
RCC1252	20	9.89 (0.28)	6.05 (0.92)	8.75 (0.71)	7.98 (0.1)	1.13 (0.1)	0.76 (0.12)	0.7 (0.16)	1.1 (0.08)
RCC1252	25	12.2 (0.21)	8.14 (0.67)	10.19 (0.75)	10.26 (0.02)	1.2 (0.1)	0.79 (0.07)	0.8 (0.02)	0.99 (0.07)
IAN01	15	10.18 (0.3)	4.07 (0.45)	9.89 (0.43)	8.31 (0.24)	1.03 (0.02)	0.49 (0.04)	0.41 (0.03)	1.19 (0.03)
IAN01	20	8.12 (0.21)	5.86 (0.11)	8.95 (0.43)	8.17 (0.04)	0.91 (0.02)	0.72 (0.01)	0.66 (0.03)	1.09 (0.05)
IAN01	25	11.21 (0.36)	6.61 (0.65)	9.95 (0.11)	8.57 (0.29)	1.13 (0.04)	0.77 (0.1)	0.66 (0.06)	1.16 (0.05)

The chemical PIC corresponds to the bulk PIC, that is the PIC in the attached and in the detached coccoliths, while the geometrical PIC corresponds to the individual cell PIC, that is the PIC in the attached coccoliths. Standard deviation of the triplicates is shown in parentheses. Temperature (*T*); coccosphere (Sph).

Table 5 lists and briefly describes the significant strain-independent and strain-specific responses to temperature found in this study.

**Table 5:** Significant strain-independent and strain-specific responses of *E*. *huxleyi* to temperature, found in this study.

Strain-independent responses	Strain-specific responses
• The number of attached coccoliths per coccosphere increased linearly with temperature (on average 2 coccoliths more each 5°C). • Coccosphere calcite mass increased linearly from 10 to 25°C, on average 12.75 pg each 5°C. • Smaller coccospheres were found at lower experimental temperatures and larger coccospheres at higher experimental temperatures. • Individual density lowest values were found at the lowest temperature treatment. • Individual sinking velocity increased linearly with temperature, from 10 to 25°C. Velocity at 25°C was of ~ 0.5 m d^-1^. • Higher numbers of detached coccoliths per cell were found at the lowest temperature treatments. • Higher numbers of total number of coccoliths per cell were found at the lowest temperature treatments.	• Cell diameter. • Coccosphere diameter. Though a positive trend with temperature is observed in the three strains.

## Discussion

Our calculated sinking rates match data based on direct measurements of another *E*. *huxleyi* strain [3]. On average, our sinking rates are by a factor of 1.3 lower than the ones reported by Bach et al. [3]. Considering that RCC1710 has a 1.2 times lower sinking rate than the other two strains (Table 3), the difference between our sinking rates and the ones reported by Bach et al. [3] could be due to strain differences. This shows that our calculations are a useful way of estimating sinking rate. In the study by Bach et al. [3], however, cells were grown at 15°C only. Therefore it remains an open question whether the response to changing temperature would show the same pattern when comparing measured and calculated sinking rates. Hence it is worthwhile to conduct a comparative study in the future, in which direct measurements are put alongside calculations as presented here.

The sinking velocity of all three strains of *E*. *huxleyi* was positively correlated to temperature. These results suggest that the effect of temperature in the sub-optimal temperature range on sinking velocity of *E*. *huxleyi* is widespread among strains isolated in different locations and moreover comparatively predictable, as indicated by the similar slopes of the linear regressions. This means that there probably is little if any clone specificity in *E*. *huxleyi*’s temperature response pattern. In total four strains were tested (one by Milner et al. [19] and three in this study), which might seem a small number but clone specificity was conspicuous when testing four clones for their responses to seawater carbonate chemistry [21]. Therefore we are confident that if responses were highly variable we would have detected that. Little inter-clone variability is a prerequisite for predicting the behaviour of natural populations. These populations are typically comprised of many different clones [33] and a relatively uniform response pattern is therefore central to any prediction. However, predicting the behaviour of natural populations on the basis of laboratory results is not straightforward for another reason. In a laboratory experiment one single physico-chemical factor can be varied and the response of a particular clone monitored. In the field, potentially many factors change simultaneously and an accurate prediction would require knowledge about the effects of all influential factors. In the course of climate change, a temperature increase will be accompanied by reduced nutrient availability and reduced seawater pH [34–36], which will be discussed below.

Our data and the ones by Milner et al. [19] show an increase in sinking rate with temperature. This effect has two components. First, a physical component which consists in the decrease in seawater density and dynamic viscosity (Table 1). Whereas the change in seawater density can account for a negligible increase in sinking rate of 0.4%, the change in dynamic viscosity causes an increase in sinking rate of ca. 28%. By contrast, our calculations yield an increase in sinking rate of ca. 70%. This difference is due to the second component, which is biological, i.e. related to changes in the individual cell density and cell size. Both individual cell density and cell size increase with temperature thus contributing to the increase in sinking rate. The changes in individual cell density are positively correlated to the individual cell PIC: POC. Therefore sinking rate correlates well with individual cell PIC: POC. This is driven by an increase in coccosphere size; not by a decrease in protoplast size. With increasing temperature there are more coccoliths in a coccosphere and the individual coccolith is heavier. In other words, the biological effect consists in the cell’s putting on more and heavier ballast stones.

**Fig 4:**
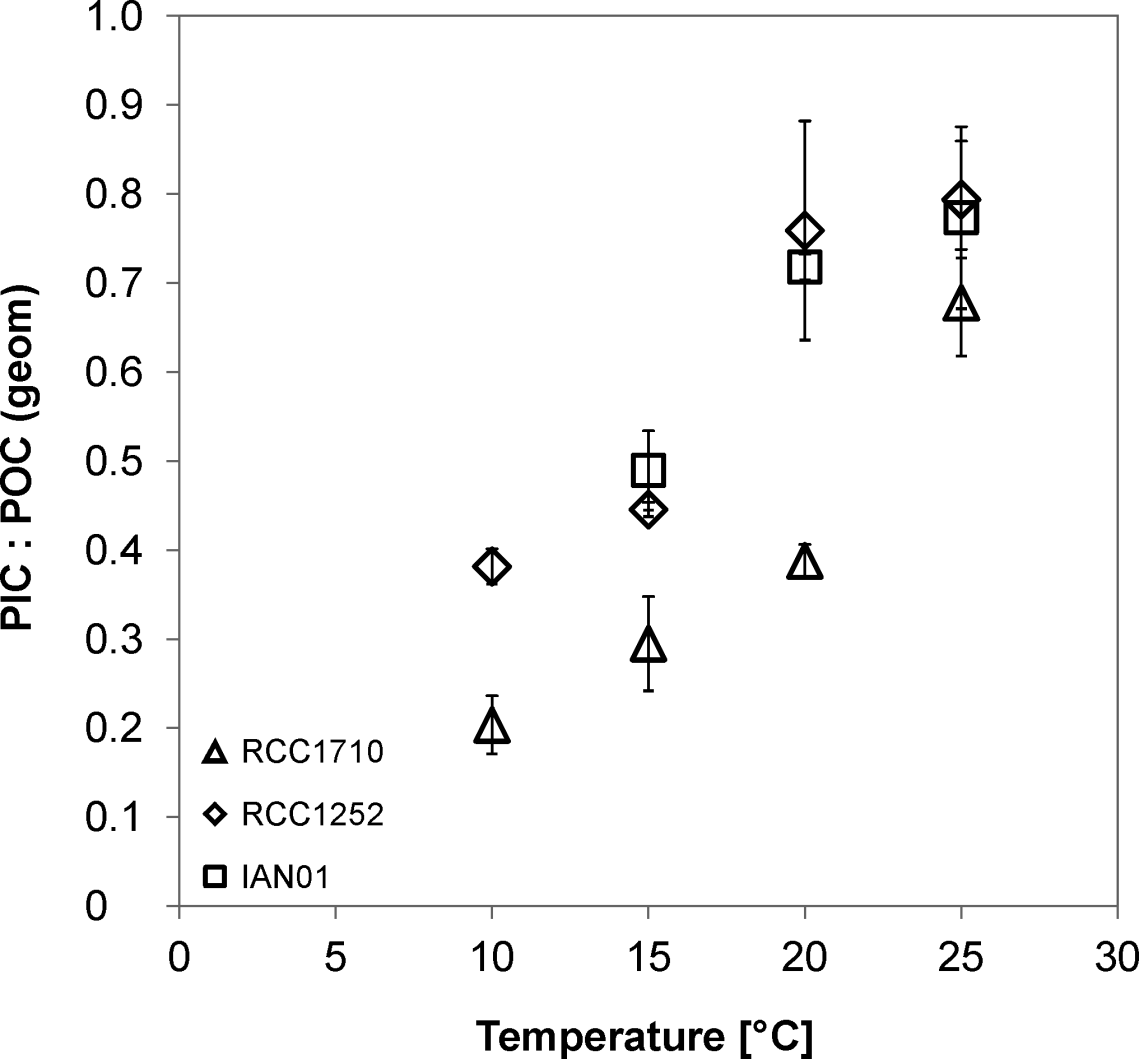
Geometrically derived ratio between the particulate inorganic carbon and the particulate organic carbon (PIC: POC) in the individual cells of three different strains of *E*. *huxleyi* grown at different temperatures. The strains are RCC1710 (triangle symbols), RCC1252 (diamond symbols), and IAN01 (square symbols). Standard deviations of the triplicate experiment results are shown.

**Fig 5:**
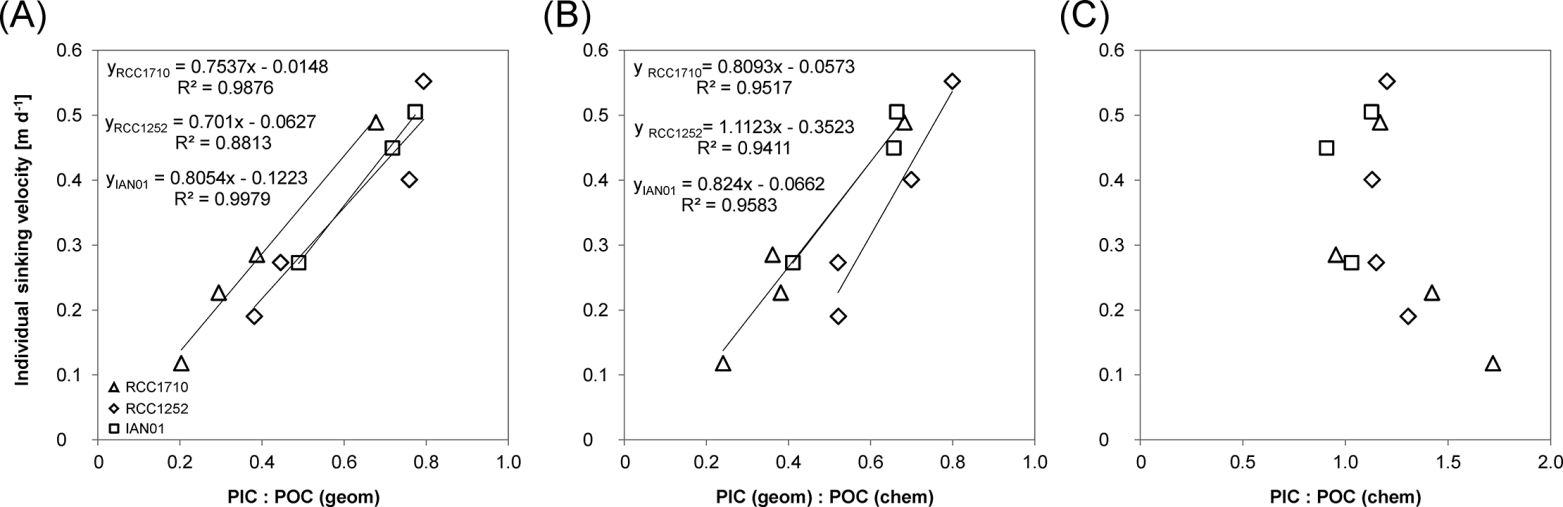
**Individual sinking velocity relationship with the geometrically derived ratio between the particulate inorganic carbon and the particulate organic carbon (PIC: POC) (A), with the geometrically derived PIC and chemically derived POC (B), and with the chemically derived PIC: POC (C).** PIC: POC in Figs A and B correspond to the individual cell ratio, while in Fig C the PIC of the ratio corresponds to the PIC in the attached plus the detached coccoliths, not only the attached as the PIC for the ratios in Figs A and B. Obtained for three different strains of *E*. *huxleyi* grown at different temperatures. Linear trend lines, corresponding equations, and r-squared values are shown in Figs A and B. The different symbols correspond to the three studied strains: strain RCC1710 (triangle symbols), strain RCC1252 (diamond symbols), and strain IAN01 (square symbols).

It is interesting to compare the bulk PIC: POC and the individual cell PIC: POC in response to temperature changes. While the bulk PIC: POC decreases with increasing temperature, the individual cell PIC: POC increases (Fig 4). This highlights the need to consider individual cell PIC: POC, not bulk PIC: POC, as an indicator of sinking velocity in *E*. *huxleyi* (Fig 5). In species which do not shed many coccoliths, e.g. *C*. *pelagicus*, this is of minor importance [8]. As an interesting methodological aside, we would like to emphasize that POC quota as determined by sample combustion tallies well with POC quota calculated from cell diameter (S2 Fig). Consequently the PIC: POC vs sinking velocity relationship holds, regardless of whether geometrically derived or directly measured POC quota is used (Fig 5). This is important because using calculated POC quota renders it possible to determine PIC: POC vs sinking velocity relationships of fossil samples such as the exceptionally well preserved material described by Gibbs et al. [37]. While the positive correlation between PIC: POC and sinking velocity seems to be robust, the correlation between PIC: POC and individual cell density is slightly less reliable. Although a positive correlation between PIC: POC and individual cell density can be seen clearly in two of our three investigated strains (Fig 2) and is also present in the one studied by Milner et al. [19] as well as in *C*. *pelagicus* [8], one strain (IAN01) shows a less convincing correlation, which was also the case in the study by Hoffmann et al. [26]. This might simply be due to too few data points.

On the whole, there seems to be a strong impact of temperature on *E*. *huxleyi* sinking rates in laboratory cultures. In the context of climate change that would mean that global warming will increase sinking rates. However, global warming does not occur in isolation but will be accompanied by seawater acidification and reduced nutrient supply. A Mediterranean *E*. *huxleyi* strain showed no change in sinking rate in response to seawater acidification [19]. Should this response be representative for *E*. *huxleyi* as a species, the influence of ocean acidification can be ignored. Nutrient limitation has long since been regarded as a calcification stimulus and it was therefore thought that ballasting and sinking rate will increase under limiting conditions [10, 38]. Recent laboratory experiments on *E*. *huxleyi* and *C*. *pelagicus*, however, showed slightly decreased sinking rates in response to macro-nutrient limitation [3, 8, 13]. If that was a general pattern, the effects of nutrient limitation and warming would work in opposite directions. The net effect observed in the field will depend partly on the local situation, because nutrient limitation is more likely in already oligotrophic areas. Hence predicting *E*. *huxleyi’*s sinking rate behaviour under climate change conditions is difficult even if only ocean acidification and nutrient limitation are considered as secondary influences (considering temperature the primary one). And of course there might be more, as yet unknown, secondary influences. Therefore it would be interesting to calculate sinking rates of different coccolithophores from field samples. This is feasible using the calculations employed here, given that coccospheres are preserved. Field studies could not only analyse extant species over seasonal and geographical temperature changes, but also fossil material over major geological climate events such as the PETM. This would provide important information on multi-factor influences in the field, and moreover include evolutionary changes in case of geological timescales. This type of information is needed for any prediction of coccolithophore sinking behaviour under future climate change.

## Conclusions

We demonstrate that the PIC: POC vs sinking velocity relationship can be determined based on geometrically derived PIC and POC quotas using raw data other than the FIB-SEM-approach [26]. This is important because FIB sectioning of coccospheres is so time consuming that it would be practically impossible to produce a dataset such as the one presented here.

Although a direct comparison between calculated and measured sinking rate using the same strain is still missing, our calculated sinking rates tally well with measured sinking rates of another strain grown at one of the temperatures used in the present study. Our sinking rate calculations are based on coccosphere architecture alone and therefore have the advantage of being applicable to field samples including fossil material. This opens up the possibility of comparative culture-field studies which are impossible using direct measurements of sinking rate. Well preserved fossil coccospheres could be used to gain information of sinking behaviour over geological timescales and major climate events such as the PETM.

Bulk PIC data includes inorganic carbon from both individual cell and loose coccoliths. The bulk PIC: POC decreases with increasing temperature but the individual cell PIC: POC increases, emphasizing the need of considering individual cell PIC: POC, not bulk PIC: POC, as an indicator of sinking velocity in *E*. *huxleyi*.

## Supporting information

S1 File Supporting information file. (PDF)

S1 Table Variables, units, and code names used in the formulas to calculate the individual sinking velocity of the coccospheres and the error propagation. (PDF)

S2 Table Average of the individual sinking velocity error propagation (prop) calculations in percentage resulted from each variable. (PDF)

S3 Table Constants or values as a function of temperature used for the calculation of sinking velocity and error propagation. Temperature (*T*).
(PDF)

S4 Table Part of the data used for the calculation of sinking velocity and error propagation. The column “Code” gives the strain name, the temperature and the bottle number. Protoplast diameter (prot diam); standard deviation (SD); standard error (SE); observed (obs); attached (att); coccolith (lith); coccosphere (sph); sample size (N).
(PDF)

S5 Table Part of the data used for the calculation of sinking velocity and error propagation and the resulted calculations. The coccolith mass refers to the coccolith calcite mass. The column “Code” gives the strain name, the temperature and the bottle number. Coccosphere (sph); diameter (diam); standard deviation (SD); standard error (SE); coccolith (lith); sample size (N); individual sinking velocity (ind sink vel); propagation (prop).
(PDF)

S6 Table Error propagation from each variable. (PDF)

S7 Table Propagation of the error in percentage from each variable. (PDF)

S1 Fig Individual sinking velocity calculation with the corresponding error propagation, for each replicate of the experiment with different *E*. *huxleyi* strains. Results are shown for strain RCC1710 (A), strain RCC1252 (B), and strain IAN01 (C), grown at different temperatures. Linear trend lines and r-squared values are shown for the calculated changes in velocity with temperature of each strain.
(PDF)

S2 Fig Cumulative probability of the particulate organic carbon (POC) ratio data. The POC ratio was calculated from the chemically derived POC and the geometrically derived POC. The plot shows that the data (circle symbols) fit a normal distribution (solid line). In a box are shown the mean and the standard deviation values with their corresponding standard errors.
(PDF)
