# Supplementary material for: Temperature effects on sinking velocity of different Emiliania huxleyi strains
Source: PLoS One. 2018 Mar 20;13(3):e0194386. doi: 10.1371/journal.pone.0194386 (PMC5860772; doi:10.1371/journal.pone.0194386)
Supplement: S1 File — (PDF) [file pone.0194386.s001.pdf]

Supporting information of

Temperature effects on sinking velocity of different *Emiliania huxleyi* strains

Rosas-Navarro et al.

Individual Sinking Velocity and Error Propagation Calculation

Here we put the formulas used to calculate the individual sinking velocity of the coccospheres and the error propagation. The units and code names used for each variable are defined in S1 Table. From seven variables in the formula, the following four correspond to laboratory measurements: number of observed attached coccoliths, protoplast diameter, coccolith calcite mass and coccosphere diameter. The experiment was performed by triplicate, each variable of each replicate presented an standard error (standard deviation divided by the square root of the sample size) which was used in the error propagation formula. The errors used for water density and dynamic viscosity (both dependent on temperature, salinity and pressure) were based on the maximum errors found for temperature and salinity during the experiment (0.5 °C and 0.5‰ respectively). The error used for the gravitational acceleration constant represents the maximum and minimum values that can be found on Earth.

Formula (F) for calculating the individual sinking velocity of coccospheres based on Stoke's law (Excel format):

```
F=(2*((PI()*4/3*(F3/2)^3*1.05+S3*I3/0.75+(PI()*4/3*(O3/2)^3
-S3*I3/0.75/2.7-PI()*4/3*(F3/2)^3)*W3/1000)/(PI()*4/3*(O3/2)^3)
-W3/1000)*$AA$3*100*((O3/(10000*2))^2))/(9*Y3*10)/100*86400
```

Simplified formula for calculating the individual sinking velocity of coccospheres (EXCEL format):

```
F=(((PI()*0.175*F3^3+S3*I3/0.75+(PI()/6*O3^3-S3*I3/0.75/2.7
-PI()*F3^3/6)*W3/1000)/PI()*6/O3^3-W3/1000)*$AA$3*100*O3^2
/200000000)*9.6/Y3
```

We can calculate the error of the F according to Courant (Courant, R.: Differential and integral calculus, vol. 2, John Wiley and Sons, 2011, page 66), as:

$$\Delta F = |\partial m_c F|\Delta m_c + |\partial d_c F|\Delta d_c + |\partial d_p F|\Delta d_p + |\partial N_c F|\Delta N_c + |\partial g F|\Delta g + |\partial \nu F|\Delta \nu + |\partial \rho F|\Delta \rho \quad \text{(A)}$$

This is valid since F and its first partial derivatives are continuous. The symbols in Eq (A) are defined in S1 Table.

Error propagation formula for each replicate (Excel Format):

```
=Z3*ABS(($AA$3*(F3^3*(0.00000504 -0.0000000048*W3)+I3*S3*
(0.0000122230996294575 - 4.52707393683613E-09*W3)))/ (O3*Y3^2))

+X3*ABS(((-0.0000000048*F3^3 - 4.52707393683613E-09*I3*S3)
*$AA$3)/(O3*Y3))

+$AB$3*ABS((F3^3*(0.00000504 -0.0000000048*W3) +I3*S3*
(0.0000122230996294575 - 4.52707393683613E-09*W3))/(O3*Y3))

+R3*ABS(($AA$3*(F3^3*(0.00000504 - 0.0000000048*W3) + I3*S3*
(0.0000122230996294575 - 4.52707393683613E-09*W3)))/(O3^2*Y3))

+3*H3*ABS((F3^2*$AA$3*(0.00000504 - 0.0000000048*W3))/(O3*Y3))

+V3*ABS((I3*$AA$3*(0.0000122230996294575 - 4.52707393683613E-09*W3))
/(O3*Y3))

+L3*ABS((S3*$AA$3*(0.0000122230996294575 - 4.52707393683613E-09*W3))
/(O3*Y3))
```

The average error propagation was of 0.038 $\pm$0.003 m d^{-1}, which resulted from the different variables in the percentages shown in S2 Table.

The individual sinking velocity calculations for each replicate of the strains RCC1710, RCC1252 and IAN01, with their corresponding error propagation, are shown in S1 Fig.

The calculated velocities and their error propagation observed in S1 Fig show a positive trend with temperature in the three strains.

In S3-S5 Tables we give the values of each measurement used in the formulas for individual sinking velocity and error propagation. The value used for the gravitational acceleration constant is 9.80665 ± 0.035 $\mathrm{m\,s^{-2}}$. In the last two columns of S5 Table we also give the results of the calculations for individual sinking velocity and error propagation. S6 Table gives the results of the error propagation from each of the variables, and S7 Table gives these same results but it percentage.

POC ratio

Particulate organic carbon (POC) quota as determined by sample combustion (i.e. chemically) tallies well with POC quota calculated from cell diameter (i.e. geometrically) (S2 Fig). S2 Fig shows that the POC ratio (ratio calculated from the chemically derived POC divided by the geometrically derived POC) is near 1 with a low standard deviation. In a cumulative probability plot, the mean value (x-axis) is placed in the 0.5 of the cumulative probability axis (y-axis).
