# Supplementary material for: Temperature effects on sinking velocity of different Emiliania huxleyi strains
Source: PLoS One. 2018 Mar 20;13(3):e0194386. doi: 10.1371/journal.pone.0194386 (PMC5860772; doi:10.1371/journal.pone.0194386)
Supplement: S1 Table — (PDF) [file pone.0194386.s002.pdf]

Variable	Unit	Symbol	Excel cell with variable value	Excel cell with standard error
Water density	[$kg\,m^{-3}$]	ρ	W3	X3
Dynamic viscosity	[$kg\,m^{-1}\,s^{-1}$]	ν	Y3	Z3
Gravitational acceleration	[$m\,s^{-2}$]	g	AA3	AB3
Observed attached coccoliths	[number]	N_c	I3	L3
Protoplast diameter	[micrometers]	d_p	F3	H3
Coccolith calcite mass	[picograms]	m_c	S3	V3
Coccosphere diameter	[micrometers]	d_c	O3	R3
