# Supplementary material for: Temperature effects on sinking velocity of different Emiliania huxleyi strains
Source: PLoS One. 2018 Mar 20;13(3):e0194386. doi: 10.1371/journal.pone.0194386 (PMC5860772; doi:10.1371/journal.pone.0194386)
Supplement: S2 Table — (PDF) [file pone.0194386.s003.pdf]

Variable	Expression	Average (SD) [%]
Error prop from water density	$\|\partial\rho F\|\Delta\rho$	1.40 (0.66)
Error prop from dynamic viscosity	$\|\partial\nu F\|\Delta\nu$	12.68 (2.50)
Error prop from gravitational acceleration	$\|\partial g F\|\Delta g$	3.33(0.56)
Error prop from observed attached coccoliths	$\|\partial N_c F\|\Delta N_c$	33.59(4.54)
Error prop from protoplast diameter	$\|\partial d_p F\|\Delta d_p$	0.20(0.11)
Error prop from coccolith mass	$\|\partial m_c F\|\Delta m_c$	30.39(7.19)
Error prop from coccosphere diameter	$\|\partial d_c F\|\Delta d_c$	18.41(2.81)
Total error propagation	ΔF	100
