# Supplementary material for: Temperature effects on sinking velocity of different Emiliania huxleyi strains
Source: PLoS One. 2018 Mar 20;13(3):e0194386. doi: 10.1371/journal.pone.0194386 (PMC5860772; doi:10.1371/journal.pone.0194386)
Supplement: S3 Table — Temperature (T). (PDF) [file pone.0194386.s004.pdf]

T [°C]	Water density [$\mathrm{kg\,m^{-3}}$]	Water density error [$\mathrm{kg\,m^{-3}}$]	Dynamic viscosity [$\mathrm{kg\,m^{-1}\,s^{-1}}$]	Dynamic viscosity error [$\mathrm{kg\,m^{-1}\,s^{-1}}$]
10	1024.611	0.472	0.0013881	0.00002110
15	1023.661	0.492	0.0012120	0.00001705
20	1022.476	0.5105	0.0010696	0.0000140
25	1021.078	0.527	0.0009524	0.00001170
