# Supplementary material for: Temperature effects on sinking velocity of different Emiliania huxleyi strains
Source: PLoS One. 2018 Mar 20;13(3):e0194386. doi: 10.1371/journal.pone.0194386 (PMC5860772; doi:10.1371/journal.pone.0194386)
Supplement: S4 Table — The column “Code” gives the strain name, the temperature and the bottle number. Protoplast diameter (prot diam); standard deviation (SD); standard error (SE); observed (obs); attached (att); coccolith (lith); coccosphere (sph); sample size (N). (PDF) [file pone.0194386.s005.pdf]

Code	Prot diam [μm]	SD	SE	Obs att lith sph^{-1}	SD	N	SE	Att lith sph^{-1}
RCC1710 10-1	4.81	1.17	0.01	12.08	3.16	100	0.32	16.11
RCC1710 10-2	4.80	1.17	0.01	11.98	2.89	60	0.37	15.98
RCC1710 10-3	4.79	1.16	0.01	13.33	4.06	60	0.52	17.78
RCC1710 15-1	5.08	1.15	0.01	14.12	4.24	34	0.72	18.83
RCC1710 15-2	5.42	1.17	0.01	13.36	3.74	54	0.51	17.82
RCC1710 15-3	5.24	1.16	0.01	14.83	5.78	54	0.79	19.78
RCC1710 20-1	4.98	1.16	0.01	15.16	4.32	46	0.63	20.21
RCC1710 20-2	4.99	1.15	0.01	14.94	4.07	51	0.57	19.92
RCC1710 20-3	5.00	1.15	0.01	15.29	4.47	48	0.65	20.39
RCC1710 25-1	4.72	1.18	0.01	16.38	4.76	39	0.77	21.84
RCC1710 25-2	4.76	1.16	0.01	16.57	4.00	49	0.57	22.10
RCC1710 25-3	4.85	1.16	0.01	16.86	5.29	49	0.76	22.48
RCC1252 10-1	4.61	1.18	0.01	13.63	3.56	57	0.47	18.18
RCC1252 10-2	4.56	1.19	0.01	12.36	4.03	61	0.52	16.48
RCC1252 10-3	4.65	1.19	0.01	12.56	5.04	61	0.65	16.74
RCC1252 15-1	4.89	1.15	0.01	13.96	4.62	47	0.67	18.61
RCC1252 15-2	4.84	1.15	0.01	14.17	3.33	52	0.46	18.90
RCC1252 15-3	4.84	1.16	0.01	14.65	3.93	52	0.54	19.54
RCC1252 20-1	4.45	1.21	0.01	15.28	5.11	47	0.75	20.37
RCC1252 20-2	4.47	1.18	0.01	15.63	6.22	52	0.86	20.85
RCC1252 20-3	4.49	1.18	0.01	14.55	4.52	55	0.61	19.39
RCC1252 25-1	4.88	1.19	0.01	17.20	5.08	51	0.71	22.93
RCC1252 25-2	4.89	1.19	0.01	16.37	4.39	49	0.63	21.82
RCC1252 25-3	4.89	1.19	0.01	17.28	5.30	49	0.76	23.04
IAN01 15-1	4.54	1.15	0.01	12.76	2.74	62	0.35	17.01
IAN01 15-2	4.58	1.15	0.01	12.73	3.31	55	0.45	16.97
IAN01 15-3	4.48	1.16	0.01	12.33	2.52	55	0.34	16.44
IAN01 20-1	4.51	1.16	0.01	13.66	3.32	53	0.46	18.21
IAN01 20-2	4.50	1.16	0.01	13.96	3.51	51	0.49	18.61
IAN01 20-3	4.51	1.16	0.01	14.12	3.39	58	0.44	18.83
IAN01 25-1	4.52	1.16	0.01	15.48	4.18	48	0.60	20.64
IAN01 25-2	4.59	1.16	0.01	15.52	3.81	50	0.54	20.69
IAN01 25-3	4.63	1.17	0.01	15.61	4.35	51	0.61	20.81
