# Supplementary material for: Temperature effects on sinking velocity of different Emiliania huxleyi strains
Source: PLoS One. 2018 Mar 20;13(3):e0194386. doi: 10.1371/journal.pone.0194386 (PMC5860772; doi:10.1371/journal.pone.0194386)
Supplement: S5 Table — The coccolith mass refers to the coccolith calcite mass. The column “Code” gives the strain name, the temperature and the bottle number. Coccosphere (sph); diameter (diam); standard deviation (SD); standard error (SE); coccolith (lith); sample size (N); individual sinking velocity (ind sink vel); propagation (prop). (PDF) [file pone.0194386.s006.pdf]

Code	Sph diam [μm]	SD	N	SE	Lith mass [pg]	SD	N	SE	Ind sink vel [$\mathrm{m\,d^{-1}}$]	Error prop [$\mathrm{m\,d^{-1}}$]
RCC1710 10-1	6.50	0.85	90	0.09	0.86	0.37	463	0.02	0.101	0.008
RCC1710 10-2	6.26	1.05	46	0.15	1.04	0.56	661	0.02	0.122	0.011
RCC1710 10-3	6.55	1.10	49	0.16	1.07	0.68	517	0.03	0.131	0.014
RCC1710 15-1	7.08	0.97	77	0.11	1.38	1.02	451	0.05	0.188	0.021
RCC1710 15-2	6.66	1.04	45	0.16	1.65	1.50	435	0.07	0.227	0.026
RCC1710 15-3	6.98	1.12	49	0.16	1.87	1.62	421	0.08	0.265	0.035
RCC1710 20-1	6.69	0.92	62	0.12	1.64	1.05	788	0.04	0.281	0.027
RCC1710 20-2	7.02	0.89	44	0.13	1.78	1.00	827	0.03	0.286	0.026
RCC1710 20-3	7.22	0.92	44	0.14	1.81	1.15	929	0.04	0.289	0.028
RCC1710 25-1	6.96	0.96	45	0.14	N/A	N/A	N/A	N/A	N/A	N/A
RCC1710 25-2	6.87	1.04	48	0.15	2.59	2.29	324	0.13	0.511	0.061
RCC1710 25-3	7.02	1.06	47	0.15	2.36	1.49	1160	0.04	0.467	0.046
RCC1252 10-1	6.67	0.73	38	0.12	1.60	1.35	350	0.07	0.188	0.021
RCC1252 10-2	5.89	0.84	79	0.09	1.61	1.82	358	0.10	0.195	0.026
RCC1252 10-3	6.25	1.11	61	0.14	1.61	1.73	325	0.10	0.187	0.027
RCC1252 15-1	6.77	0.99	39	0.16	2.05	1.01	325	0.06	0.277	0.031
RCC1252 15-2	6.87	0.75	52	0.10	1.93	1.50	322	0.08	0.262	0.028
RCC1252 15-3	6.59	0.84	52	0.12	1.93	1.33	327	0.07	0.281	0.030
RCC1252 20-1	7.03	1.24	48	0.18	2.65	2.20	379	0.11	0.417	0.055
RCC1252 20-2	6.80	1.20	53	0.16	2.67	2.54	406	0.13	0.443	0.062
RCC1252 20-3	6.64	0.96	55	0.13	2.14	2.10	376	0.11	0.343	0.043
RCC1252 25-1	7.46	1.02	48	0.15	2.91	1.94	330	0.11	0.548	0.061
RCC1252 25-2	7.60	1.16	50	0.16	2.88	2.59	430	0.13	0.508	0.059
RCC1252 25-3	7.49	1.19	43	0.18	3.21	2.69	365	0.14	0.601	0.076
IAN01 15-1	6.29	0.90	48	0.13	2.17	1.77	350	0.09	0.285	0.031
IAN01 15-2	5.94	0.78	54	0.11	2.07	1.27	337	0.07	0.289	0.029
IAN01 15-3	5.97	0.64	55	0.09	1.81	0.65	323	0.04	0.245	0.019
IAN01 20-1	6.04	0.72	54	0.10	2.63	1.69	418	0.08	0.432	0.042
IAN01 20-2	5.91	0.86	45	0.13	2.63	1.61	360	0.08	0.452	0.047
IAN01 20-3	5.82	0.71	57	0.09	2.64	0.79	352	0.04	0.465	0.037
IAN01 25-1	7.10	1.02	47	0.15	2.93	2.19	355	0.12	0.519	0.059
IAN01 25-2	6.51	0.99	49	0.14	2.66	2.35	386	0.12	0.517	0.060
IAN01 25-3	6.37	0.90	51	0.13	2.39	2.14	366	0.11	0.480	0.057
