# Supplementary material for: Temperature effects on sinking velocity of different Emiliania huxleyi strains
Source: PLoS One. 2018 Mar 20;13(3):e0194386. doi: 10.1371/journal.pone.0194386 (PMC5860772; doi:10.1371/journal.pone.0194386)
Supplement: S6 Table — (PDF) [file pone.0194386.s007.pdf]

Code	$\lvert\partial\rho F\rvert\Delta\rho$ [$\mathrm{m\,d^{-1}}$]	$\lvert\partial\nu F\rvert\Delta\nu$ [$\mathrm{m\,d^{-1}}$]	$\lvert\partial g F\rvert\Delta g$ [$\mathrm{m\,d^{-1}}$]	$\lvert\partial N_c F\rvert\Delta N_c$ [$\mathrm{m\,d^{-1}}$]	$\lvert\partial d_p F\rvert\Delta d_p$ [$\mathrm{m\,d^{-1}}$]	$\lvert\partial m_c F\rvert\Delta m_c$ [$\mathrm{m\,d^{-1}}$]	$\lvert\partial d_c F\rvert\Delta d_c$ [$\mathrm{m\,d^{-1}}$]
RCC1710 10-1	0.000	0.002	0.000	0.002	0.000	0.002	0.001
RCC1710 10-2	0.000	0.002	0.000	0.003	0.000	0.002	0.003
RCC1710 10-3	0.000	0.002	0.000	0.005	0.000	0.003	0.003
RCC1710 15-1	0.000	0.003	0.001	0.009	0.000	0.006	0.003
RCC1710 15-2	0.001	0.003	0.001	0.008	0.000	0.009	0.005
RCC1710 15-3	0.000	0.004	0.001	0.013	0.000	0.010	0.006
RCC1710 20-1	0.000	0.004	0.001	0.011	0.000	0.006	0.005
RCC1710 20-2	0.000	0.004	0.001	0.010	0.000	0.005	0.005
RCC1710 20-3	0.000	0.004	0.001	0.011	0.000	0.006	0.006
RCC1710 25-2	0.001	0.006	0.002	0.017	0.000	0.024	0.011
RCC1710 25-3	0.001	0.006	0.002	0.020	0.000	0.008	0.010
RCC1252 10-1	0.000	0.003	0.001	0.006	0.000	0.008	0.003
RCC1252 10-2	0.000	0.003	0.001	0.008	0.000	0.011	0.003
RCC1252 10-3	0.000	0.003	0.001	0.009	0.000	0.010	0.004
RCC1252 15-1	0.000	0.004	0.001	0.013	0.000	0.007	0.006
RCC1252 15-2	0.000	0.004	0.001	0.008	0.000	0.011	0.004
RCC1252 15-3	0.000	0.004	0.001	0.010	0.000	0.010	0.005
RCC1252 20-1	0.000	0.005	0.001	0.020	0.000	0.017	0.011
RCC1252 20-2	0.000	0.006	0.002	0.024	0.000	0.020	0.011
RCC1252 20-3	0.000	0.004	0.001	0.014	0.000	0.016	0.007
RCC1252 25-1	0.001	0.007	0.002	0.022	0.000	0.019	0.011
RCC1252 25-2	0.001	0.006	0.002	0.019	0.000	0.021	0.011
RCC1252 25-3	0.001	0.007	0.002	0.025	0.000	0.025	0.015
IAN01 15-1	0.000	0.004	0.001	0.007	0.000	0.012	0.006
IAN01 15-2	0.000	0.004	0.001	0.010	0.000	0.009	0.005
IAN01 15-3	0.000	0.003	0.001	0.006	0.000	0.005	0.004
IAN01 20-1	0.000	0.006	0.002	0.014	0.000	0.013	0.007
IAN01 20-2	0.000	0.006	0.002	0.015	0.000	0.014	0.010
IAN01 20-3	0.000	0.006	0.002	0.014	0.000	0.007	0.008
IAN01 25-1	0.000	0.006	0.002	0.020	0.000	0.020	0.011
IAN01 25-2	0.001	0.006	0.002	0.017	0.000	0.022	0.011
IAN01 25-3	0.001	0.006	0.002	0.018	0.000	0.021	0.009
