# Supplementary material for: Temperature effects on sinking velocity of different Emiliania huxleyi strains
Source: PLoS One. 2018 Mar 20;13(3):e0194386. doi: 10.1371/journal.pone.0194386 (PMC5860772; doi:10.1371/journal.pone.0194386)
Supplement: S7 Table — (PDF) [file pone.0194386.s008.pdf]

Code	$\lvert\partial\rho F\rvert\Delta\rho$ [%]	$\lvert\partial\nu F\rvert\Delta\nu$ [%]	$\lvert\partial g F\rvert\Delta g$ [%]	$\lvert\partial N_c F\rvert\Delta N_c$ [%]	$\lvert\partial d_p F\rvert\Delta d_p$ [%]	$\lvert\partial m_c F\rvert\Delta m_c$ [%]	$\lvert\partial d_c F\rvert\Delta d_c$ [%]	Total [%]
RCC1710 10-1	3.92	20.14	4.73	29.57	0.63	22.70	18.32	100
RCC1710 10-2	2.79	16.54	3.88	29.66	0.44	19.89	26.80	100
RCC1710 10-3	2.18	14.46	3.39	33.29	0.34	23.44	22.90	100
RCC1710 15-1	1.89	12.41	3.15	40.71	0.27	27.80	13.77	100
RCC1710 15-2	1.95	12.07	3.06	29.20	0.27	33.40	20.05	100
RCC1710 15-3	1.35	10.80	2.74	37.50	0.18	29.76	17.67	100
RCC1710 20-1	1.83	13.68	3.73	40.29	0.26	21.87	18.33	100
RCC1710 20-2	1.84	14.40	3.93	38.81	0.26	19.82	20.95	100
RCC1710 20-3	1.70	13.62	3.71	40.73	0.23	20.10	19.90	100
RCC1710 25-2	0.93	10.34	3.01	27.76	0.12	39.53	18.31	100
RCC1710 25-3	1.21	12.36	3.59	42.89	0.16	17.67	22.11	100
RCC1252 10-1	1.34	13.52	3.17	28.73	0.21	37.20	15.82	100
RCC1252 10-2	1.21	11.62	2.73	29.62	0.19	42.34	12.28	100
RCC1252 10-3	1.12	10.41	2.44	32.61	0.17	37.73	15.51	100
RCC1252 15-1	1.30	12.41	3.15	39.90	0.18	22.51	20.56	100
RCC1252 15-2	1.40	13.34	3.38	28.90	0.19	38.43	14.36	100
RCC1252 15-3	1.34	13.10	3.32	32.43	0.19	33.18	16.44	100
RCC1252 20-1	0.74	9.96	2.72	35.78	0.10	31.27	19.44	100
RCC1252 20-2	0.68	9.31	2.54	37.80	0.09	32.37	17.21	100
RCC1252 20-3	0.94	10.45	2.85	31.81	0.14	38.34	15.48	100
RCC1252 25-1	0.93	11.01	3.20	35.56	0.12	31.48	17.70	100
RCC1252 25-2	0.93	10.52	3.06	31.36	0.12	35.57	18.43	100
RCC1252 25-3	0.78	9.77	2.84	33.53	0.10	33.65	19.33	100
IAN01 15-1	1.19	13.15	3.34	24.13	0.17	38.61	19.42	100
IAN01 15-2	1.32	13.86	3.52	32.55	0.19	31.00	17.56	100
IAN01 15-3	1.86	17.98	4.56	32.97	0.28	23.98	18.37	100
IAN01 20-1	1.12	13.61	3.71	33.19	0.15	31.35	16.87	100
IAN01 20-2	1.01	12.56	3.43	32.40	0.14	29.60	20.86	100
IAN01 20-3	1.32	16.44	4.48	37.95	0.18	19.23	20.39	100
IAN01 25-1	0.84	10.80	3.14	33.06	0.11	33.63	18.42	100
IAN01 25-2	0.91	10.65	3.09	28.87	0.12	37.47	18.89	100
IAN01 25-3	0.96	10.33	3.00	31.32	0.13	37.64	16.61	100
