# Supplementary figures and images for: Temperature effects on sinking velocity of different Emiliania huxleyi strains
Source: PLoS One. 2018 Mar 20;13(3):e0194386. doi: 10.1371/journal.pone.0194386 (PMC5860772; doi:10.1371/journal.pone.0194386)

(A)

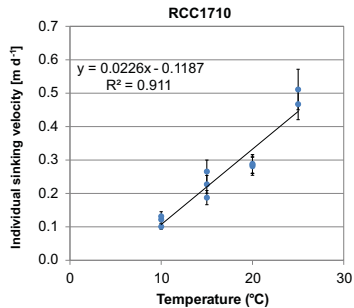

(B)

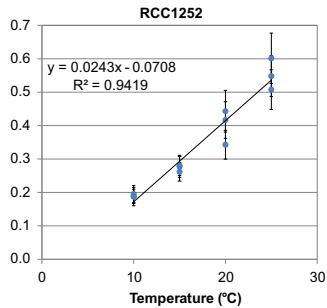

(C)

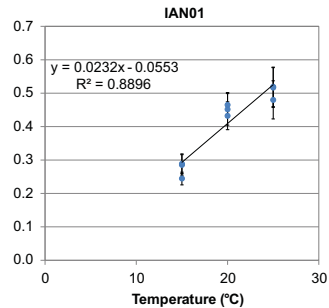

Supplement: S1 Fig — Results are shown for strain RCC1710 (A), strain RCC1252 (B), and strain IAN01 (C), grown at different temperatures. Linear trend lines and r-squared values are shown for the calculated changes in velocity with temperature of each strain. (PDF) [file pone.0194386.s009.pdf]

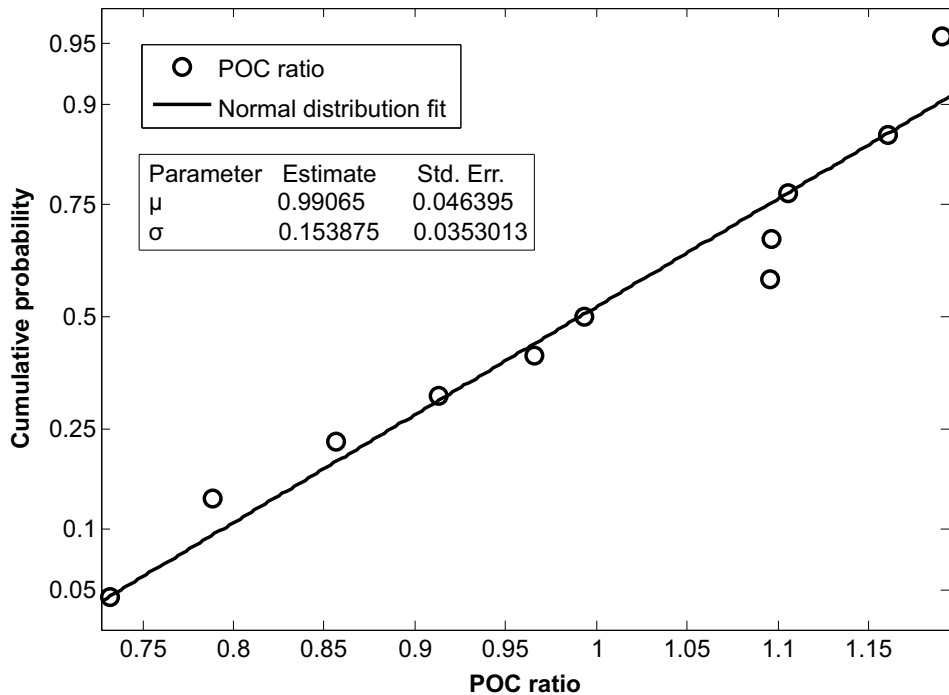

Supplement: S2 Fig — The POC ratio was calculated from the chemically derived POC and the geometrically derived POC. The plot shows that the data (circle symbols) fit a normal distribution (solid line). In a box are shown the mean and the standard deviation values with their corresponding standard errors. (PDF) [file pone.0194386.s010.pdf]
